# Management of Nonceliac Gluten Sensitivity by Gastroenterology Specialists: Data from an Italian Survey

**DOI:** 10.1155/2015/530136

**Published:** 2015-11-19

**Authors:** Federica Branchi, Francesca Ferretti, Lorenzo Norsa, Leda Roncoroni, Dario Conte, Maria Teresa Bardella, Luca Elli

**Affiliations:** ^1^Center for The Prevention and Diagnosis of Celiac Disease, Gastroenterology and Endoscopy Unit, Fondazione IRCCS Cà Granda Ospedale Maggiore Policlinico, Via F. Sforza 35, 20100 Milan, Italy; ^2^Department of Pathophysiology and Transplantation, Università degli Studi di Milano, Via F. Sforza 35, 20100 Milan, Italy

## Abstract

*Background and Aim*. Nonceliac gluten sensitivity is syndrome characterized by symptoms disappearing after a gluten-free diet. Its existence is still argument of discussion among specialists. Our aim was to evaluate the knowledge about nonceliac gluten sensitivity among gastroenterology specialists.* Methods*. During October 2013 a questionnaire was sent through a medical newsletter to Italian gastroenterologists. Twelve questions investigated their knowledge on nonceliac gluten sensitivity, including their diagnostic and therapeutic approach.* Results*. A total of 212 gastroenterologists filled in the questionnaire. The 98.6% were aware of the existence of a syndrome called “nonceliac gluten sensitivity” and 77% believe in its existence. However, only 56% gave a correct definition of the term. The majority of specialists diagnosed gluten sensitive patients and the number of diagnoses was not statistically different from that of celiac disease. Moreover, a gluten-free diet was prescribed by 64% of the specialists and among them the 73% noted an increase of gluten sensitive patients attending their outpatient services.* Conclusions*. Our study indicated that most of the specialists recognize nonceliac gluten sensitivity and prescribe gluten-free diet, although 44% of the specialists are not able to give its correct definition; underlining the necessity of medical education on this topic is needed.

## 1. Introduction

Gluten-related disorders include a spectrum of clinical conditions associated with dietary exposure to gluten [1–3]. Historically, celiac disease (CD), gluten ataxia, and dermatitis herpetiformis were the only gluten-related diseases, but the current clinical scenario is more diversified, making the diagnostic work-up challenging for physicians [4]. Nowadays, there is increasing awareness on the importance of ruling out forms of wheat allergy (WA) when facing with unclear cases of suspected gluten-related disorders [2, 3]. In addition, attention has been focused on a relatively new clinical entity labeled as nonceliac gluten sensitivity (NCGS); NCGS is a heterogeneous disorder induced by gluten ingestion in which neither allergic nor autoimmune mechanisms seem to be involved [5, 6]. The clinical spectrum of NCGS has been reported to range from upper/lower gastrointestinal symptoms to extraintestinal symptoms, including headache, malaise, asthenia, and muscle cramps [7].

The first report about a syndrome caused by exposure to gluten in absence of CD dates back to 1978 [8–10], but for decades this clinical entity was set apart [2]. Actually, data from epidemiological studies reveal that patients refer to gastroenterologists and other specialists for a wide range of gluten-related symptoms, even in the absence of a definite diagnosis of CD or WA [11–13]. Moreover, in the last few years the self-prescription of GFD has emerged as a relevant phenomenon, apparently based on the perception of being a “healthier diet” [14, 15].

To date, international consensus has been reached for a shared definition of NCGS and its characteristics [2, 3, 16]. However, some controversial aspects are still in need of clarification. First of all, the lack of specific biomarkers for the identification of NCGS makes the diagnostic approach difficult. For this reason, CD and WA always have to be ruled out before considering the hypothesis of NCGS. A stepwise algorithm has been proposed to help physicians in the diagnostic approach to patients with suspected NCGS, with the aim of differentiating NCGS from CD, especially in subjects already on a GFD [17]. Moreover, an overlap of symptoms between the irritable bowel syndrome (IBS) and NCGS has been reported [18, 19] and a nonspecific symptomatic improvement in patients with suspected NCGS has been described with a diet poor in low-fermentable, poorly absorbed, short-chain carbohydrates (FODMAPs) [20], so that even the existence of NCGS has been put into questioning [19–21].

In everyday clinical practice, continuous updates and training of the physicians are mandatory. To date, it is not clear if gastroenterologists receive adequate information regarding the state of the art in the field of gluten-related disorders and to the more correct diagnostic approach to patients suspected of NCGS. With these premises, the aim of our study was to assess the awareness and knowledge of NCGS and gluten-related disorders among Italian gastroenterologists.

## 2. Methods

### 2.1. Questionnaire and Participants

This cross-sectional study was designed to assess the degree of knowledge and awareness about gluten-related disorders and NCGS among Italian gastroenterologists. The survey was proposed to Italian gastroenterology specialists working in public hospitals through an anonymous questionnaire attached to a medical newsletter (“Panorama Medico” edited by Merqurio). The newsletter was sent in October 2013 for a total of three times during the month in order to improve adherence to the survey.

The questionnaire consisted of 12 multiple-choice questions inquiring about NCGS (nonceliac gluten sensitivity) and CD (celiac disease) as follows.

*Details of the Questionnaire Presented to Italian Gastroenterologists*

1. When did you first hear about NCGS?
  - More than three years ago
  - From three to two years ago
  - In the last 12 months
  - I do not remember when I heard about NCGS
  - I do not know what NCGS is
2. What is the definition of NCGS?
  - It is a synonym of celiac disease
  - It is a syndrome characterized by symptoms associated to gluten ingestion in absence of allergy and celiac disease
  - It is a general term indicating all the gluten-related disorders
  - I heard about NCGS but I do not know its definition
  - I do not know
3. What is your opinion on NCGS?
  - It is a serious and real disorder
  - It is a real entity and I already follow NCGS patients
  - I'm not sure about the existence of NCGS
  - I think NCGS is only a matter of fashion
4. Beside gastroenterologists, do you think other specialists are involved in the management of patients with NCGS?
  - General Practitioner
  - Dietician
  - Pharmacist
  - Allergologist
  - Others
5. How many NCGS patients have you diagnosed in the last 12 months?
  - 0
  - 1–10
  - 11–25
  - 26–50
  - 51–100
  - >100
6. How many CD patients have you diagnosed in the last 12 months?
  - 0
  - 1–10
  - 11–25
  - 26–50
  - 51–100
  - >100
7. Estimate the possible prevalence of NCGS in your region
  - 1 : 20
  - 1 : 100
  - 1 : 250
  - 1 : 500
  - 1 : 1000
  - I do not know
8. Estimate the prevalence of CD in your region
  - 1 : 20
  - 1 : 100
  - 1 : 250
  - 1 : 500
  - 1 : 1000
  - I do not know
9. Once you diagnose a patient with NCGS, what do you prescribe?
  - A gluten-free diet
  - A dietician counseling
  - A gluten-free diet and I prepared by my self some flyers about this type of diet
10. What are your main sources of information for updates on CD and NCGS?
  - Material from the gluten-free companies
  - Scientific studies
  - Journals, newspapers, internet, and so forth
11. In your opinion, is the incidence of NCGS increasing?
  - Yes
  - No
  - I do not know
12. If so, could it be for a specific reason?
  - The patients are aware about the NCGS and consider GFD healthy
  - Physicians are more informed about NCGS
  - A general awareness about NCGS
  - Patients' associations are more sensible to the existence of NCGS patients

In addition, participants should provide supplementary personal information (age, sex, fields of expertise, and area/region of provenance). In the questionnaire, CD and NCGS definitions were given following the international guideline [2, 22].

The study was in line with the Italian law on privacy matter and was carried out in accordance with the declaration of Helsinki.

### 2.2. Statistical Analysis

All the assumptions were verified by GraphPad prism software version 5.0 (GraphPad Software, San Diego, California, USA), and a *p* value < 0.05 was considered as statistically significant (test significance level: 5%, two tails). Categorical variables were compared by *χ* ^2^ or Fisher's exact tests. Kolmogorov-Smirnov's test was used to assess normal distribution. Correlations were analysed by Pearson or Spearman's tests.

## 3. Results

Out of 1322 gastroenterology specialists asked to take part in the survey, a total of 212 (16%) adhered and filled in the questionnaire. The participant cohort consisted of 186 males (88%) and 26 (12%) females, with a median age of 55 years (range 30–68); the majority were expert gastroenterologists (78% older than 50 years old and with more than 20 years of clinical practice). Participants worked in gastroenterology or internal medicine units all over Italy (public hospitals), with a homogeneous distribution between geographical areas: 79 (37%) worked in Southern Italy, 70 (33%) in Northern Italy, and 63 (30%) in Central Italy. Almost all of the participants reported to be familiar with the term NCGS for more than 3 years, with only 3 of them (1.4%) unaware of the existence of this definition. However, when asked about this term, 119 (56.1%) correctly associated the term NCGS with a clinical entity different from CD and WA; 58 (27.4%) of the participants identified NCGS as a nonspecific umbrella term, 19 (9%) considered NCGS as a synonym of WA, and 16 (7.5%) were unable to answer. No statistical difference was found between the group correctly defining the NCGS and the other groups. In spite of a certain degree of inappropriate use of the definition, 164 (77.3%) specialists considered NCGS a clinical condition worthy of attention, while 31 (14.7%) questioned the existence of NCGS and 17 (8%) reported a skeptical attitude towards this entity. As expected, the main sources of information about NCGS were the scientific publications for almost all of the participants.

**Figure 1:**
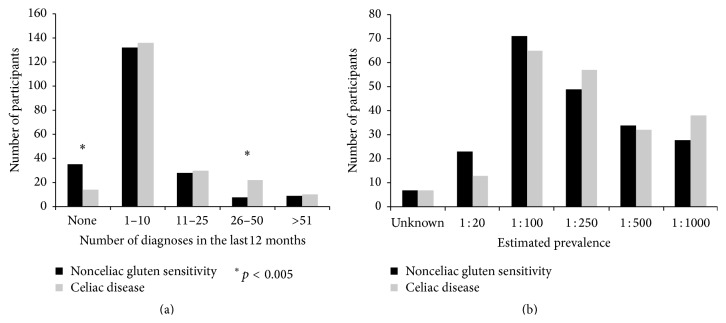
Number of diagnoses of nonceliac gluten sensitivity and celiac disease reported by participants in 12 months (a). Prevalence of nonceliac gluten sensitivity and celiac disease as estimated by participants (b).

The majority of the participants reported to have dealt with at least 1 diagnosis of NCGS within the previous 12 months, with 62% reporting from 1 to 10 diagnoses. The distribution of NCGS and CD diagnosis among participants is shown in Figure 1(a). Interestingly, the distribution of participants reporting 0 (17%) versus 1–10 (62%) versus 11–25 (13%) versus >26 (8%) diagnoses of NCGS per year was similar to the distribution of new diagnoses of CD, probably reflecting a more frequent contact with patients with gluten-related disorders in outpatient clinics already managing CD.

The participants were also asked to give an estimated prevalence of NCGS. As expected in the setting of a relatively new clinical entity, there was low agreement among gastroenterology specialists. In Figure 1(b) the reported estimated prevalence of NCGS as compared to CD in Italy is detailed. Interestingly, 73% of the participants observed an increase of referral to their outpatient clinics due to gastrointestinal and extraintestinal symptoms consistent with/attributed to NCGS. Improvement of medical education on NCGS is considered the main reason of this increase (75.4%); however, 25% ascribed this data to the spread of beliefs about the possible benefit of the GFD.

According to our survey, the management of NCGS by gastroenterology specialist mostly consists in prescribing a gluten-free diet (62%) once the diagnosis has been made (i.e., CD and wheat allergy have been excluded) while 22% of the participants reported to prescribe an evaluation by a dietician and 16% declared to discuss the matter with the patient with the aid of scientific or informative material. Participants reported that general physicians and dieticians were frequently involved along with the gastroenterologists in the diagnosis and management of patients with NCGS.

Age and sex of the specialists did not influence the answers to the questionnaire.

## 4. Discussion

The results of this survey show that Italian gastroenterology specialists are aware of the advances in the scenario of gluten-related disorders and actively consider NCGS in the differential diagnosis, prescribing a GFD also in the absence of CD or WA. However, 40% are not able to give a correct definition of NCGS underlining the need for continuous medical education.

The recent increase of scientific publications in the field of gluten-related disorders (such as the attention of the media and market) could explain why more than three-quarters of Italian gastroenterologists are informed about the existence of NCGS for more than three years, but only half of them were able to define the term correctly. Some degree of confusion is coherent with the fact that a consensus on the classification of gluten-related disorders and on an “official” definition of NCGS has been reached only lately [1]. To date, NCGS is still a diagnosis of exclusion, achieved after other disorders have been ruled out, and only recent publications introduced active criteria [3, 17]. Moreover, the spectrum of symptoms attributed to NCGS shows some overlap with IBS, so that the double blind placebo controlled gluten challenge is necessary to differentiate the two conditions. A study of Carroccio et al. [23] suggested that approximately one-third of IBS patients could be affected by multiple food hypersensitivity, including wheat sensitivity.

This kind of uncertainty surrounding a relatively new clinical entity could be considered responsible for the criticism and skeptical attitude toward NCGS in the medical community [24–26], which was reflected in the answer of almost one-third of the participants to our survey.

Moreover, given the dramatic increase in scientific publications on NCGS and the growing attention of the media on these subjects, it could be questioned whether the “birth” of NCGS could be the result of the so-called “gold effect,” which refers to the circumstance when a scientific idea reaches the status of an accepted truth within a professional community by means of conferences, committees, and consensus building, despite not being supported by conclusive evidence [27]. However, in spite of data questioning the consistency of NCGS as a clinical entity [20, 28], increasing evidence of its existence is emerging from recent studies and efforts are headed towards the establishment of clear diagnostic criteria [3, 28].

The epidemiological data from our questionnaire suggested similar rates of CD and NCGS diagnosis as reported by Italian gastroenterologists. It is an interesting observation but further data are needed in the light of the current epidemiological knowledge on NCGS [15]. A quarter of the participants reported an increased rate of NCGS, which they attributed to patients awareness and attention to gluten-related disorders and GFD, also resulting in higher rates of self-prescribed GFD [29].

According to 70% of participants of our survey, patients with suspected NCGS first refer to their general practitioners, who probably have less familiarity with this condition and its management, with a high risk of a failure in excluding CD and WA.

An important limitation of this study is the adhesion to the survey, which could also imply the selection of specialists particularly interested in this topic. Moreover, the Italian health system constituted by public hospitals and the presence of an economic support for the access to gluten-free products in case of CD diagnosis should be considered. This latter could increase the attention of physician and patients for gluten-related disorders. Still, the collected data give a reliable overview of the change in the scenario of gluten-related disorders and functional disorders experienced by our scientific community. The therapeutic approach of patients with NCGS, involving the prescription of a GFD even in the absence of CD, was reportedly considered by the wide majority of the participants to the survey. Nevertheless, many of them declared not to employ specific information material or provide contact with a dietitian. These results could be explained by the lack of dietetics services in many Italian hospitals.

## 5. Conclusion

NCGS has become a topic of increasing interest for the scientific community. The results of this survey demonstrate a certain degree of awareness of the clinical relevance of gluten-related disorders among Italian gastroenterology specialists. Despite this, 40% of the survey responders are not able to give the correct definition of NCGS. Efforts should be made to improve the knowledge about these disorders, as well as promote the circulation of unambiguous and scientifically relevant information. The establishment of new guidelines approved by the international scientific community would help towards the proper management of patients with gluten-related disorders.
